# Explainable machine learning framework for foodborne disease outbreak prediction in Eastern Province, Saudi Arabia: case study

**DOI:** 10.3389/fpubh.2026.1883871

**Published:** 2026-08-21

**Authors:** Naof Faiz Saleem Al-Ansary, Mahmoud Berekaa, Raghad Alhotheyfa, Abdullah Almharfi, Megha Arakeri, Tusar Kanti Mishra

**Affiliations:** 1 Department of Public Health, College of Public Health, Imam Abdulrahman bin Faisal University, Dammam, Saudi Arabia; 2 Department of Environmental Health, College of Public Health, Imam Abdulrahman bin Faisal University, Dammam, Saudi Arabia; 3 Eastern Region Municipality, Dammam, Saudi Arabia; 4 Manipal Institute of Technology Bengaluru, Manipal Academy of Higher Education, Manipal, India

**Keywords:** anomaly detection, explainable AI, foodborne disease, graph-based modeling, large language model, public health, SHAP, XGBoost

## Abstract

The occurrence of foodborne diseases is a considerable public health issue, especially in areas that are quickly becoming urbanized with intricate food delivery systems. In this paper, we present a machine learning-based model for predicting outbreaks, explainability, and spatial risk propagation, validated through a multiyear data set of an epidemiological nature from 12 cities in the Eastern Province of Saudi Arabia (2021–2025). The final data set includes 61 cases and 13 engineered features. In the current research, the proposed architecture uses XGBoost to predict outbreaks, alongside using the random forest for predicting severity and support vector machine (SVM) for comparisons. The XGBoost classifier demonstrates an evenly balanced performance (accuracy = 0.85, precision = 0.78, recall = 0.78) on the testing set. Due to the size of the dataset, the results are provided with the estimation of uncertainty (rather than the exact numbers). Using leakage-safe repeated stratified cross-validation, the mean AUC equals 0.64 [95% interval = (0.20, 1.00)], and the leave-one-year-out validation method is not stable (mean AUC 0.47). Differences between the models (e.g., better single-split cross-validation AUC for SVM) are within confidence intervals. Interpretability is improved by using the SHAP framework to measure feature importance, which shows that the main factors are hospitalization and the severity of symptoms. The graph module also helps in understanding the propagation of disease risk between cities, highlighting the importance of well-connected metropolitan areas as disease hubs. Moreover, the use of a locally deployed Mistral LLM makes the generated explanations more readable. The findings show that our approach presents an appropriate balance of predictiveness, interpretability, and spatial knowledge. With only 61 data points and 11 outbreaks reported, this research is clearly not meant to be an early warning system, but rather a proof-of-concept on how one might be designed. In order to ensure reproducibility, the preprocessing pipeline and synthetic dataset generator have been made available to the community.

## 1 Introduction

Contamination of food with unsafe food products has been cited as one of the major causes of foodborne diseases. These diseases range from minor gastrointestinal ailments like diarrhea to serious ailments including cancers, which are major threats to human life. Foodborne diseases are caused by a variety of contaminants such as bacteria, viruses, fungi, and parasites spread through contaminated food (1). Although preventable, foodborne diseases remain a global public health problem. As indicated by WHO, 600 million people suffer from contaminated foods each year leading to about 420,000 deaths globally (2). Apart from this, surveillance data obtained from the Center for Disease Control and Prevention indicates about 23,610 foodborne illness outbreaks in 2023, which accounted for about 449,405 illnesses, 20,925 hospitalizations, and 510 deaths. Several researches conducted previously indicated increased rates of hospitalizations and mortality attributed to foodborne diseases (3, 4). Similarly, Scallan et al. (5) indicated that there are about 47.8 million cases, 127,839 hospitalizations, and 3,037 deaths each year in the United States due to foodborne diseases.

In the case of Saudi Arabia, some of the factors leading to an increase in foodborne disease outbreaks include harsh climatic conditions, fast urbanization, complicated food chains, and huge events such as Hajj pilgrimage (6–9). These factors result in a situation where foodborne disease outbreaks have a chance of occurring. The conventional way of identifying and responding to outbreaks is through manual reporting and laboratory testing, which takes a lot of time and is not adequate for dealing with outbreaks. Therefore, there is a need for an efficient approach for the early detection of foodborne disease outbreaks. Machine learning (ML) has proven an efficient tool in examining and predicting foodborne disease outbreaks due to its ability to detect trends in complex data sets and enable the choice of relevant predictive variables (10). The efficacy of artificial intelligence in food safety monitoring has been shown in a number of studies recently; however, there is still little research on detecting and predicting foodborne pathogens and outbreaks (11–15). Moreover, progress in ML and deep learning has allowed increasing the accuracy of detection, decreasing response time, and automating food safety procedures (11, 16).

**Figure 1:**
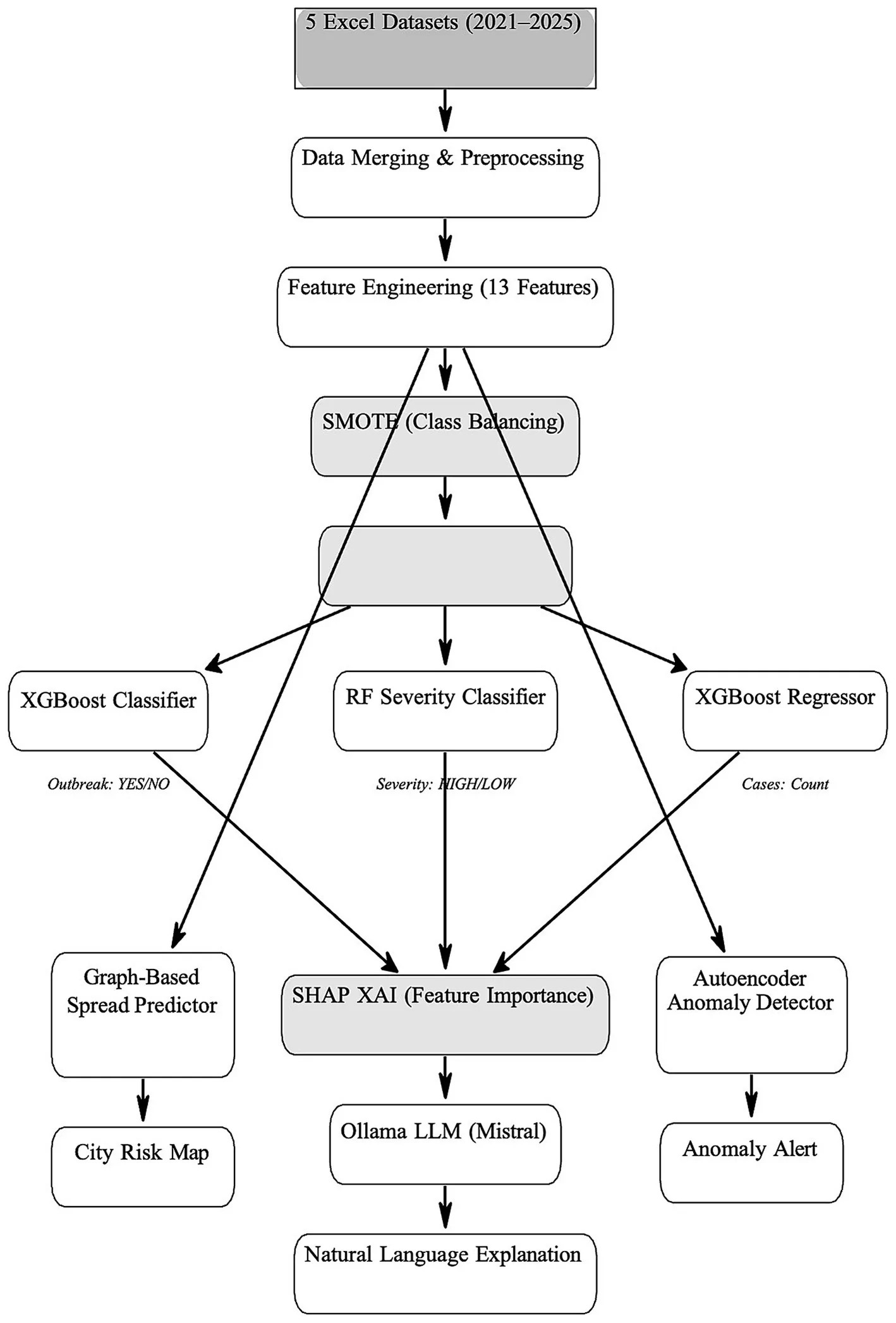
Overall system architecture.

In Saudi Arabia, the most recent epidemiological studies show a decrease in the total number of outbreaks from 2017 until 2023; however, there are some pathogens that are quite common, namely *Salmonella* spp. and *Entamoeba* spp. Public health agencies in Saudi Arabia keep working on detection of causes and locations of outbreaks (17). Notwithstanding all these improvements, one key drawback that the existing machine learning algorithms possess in the field of healthcare can be described as the “black box” problem, which makes them less interpretable and trustworthy when used in decision making. In order to solve the mentioned issue, this research aims at developing a hybrid solution that will combine XGBoost-based outbreak prediction algorithm, SHAP-based explainable artificial intelligence (XAI) technique, locally running large language model (LLM) used for creating human-readable explanations, and graph-based component responsible for modeling spatial outbreak propagation. In addition to achieving higher levels of prediction accuracy, such an approach will allow for improving interpretability and spatial awareness of the decision-making process. This study will employ an applied, quantitative research design that will be based on retrospectively collected epidemiological data. As a methodological framework, hybrid approach, incorporating supervised machine learning, explainable AI (SHAP), unsupervised anomaly detection, and graph-based spatial analysis will be applied. Overall system architecture is presented in Figure 1.

At the very beginning of this study, we would like to stress the fact that due to the small amount of data (61 cases, 11 outbreaks), this analysis is purely explorative and shows how such methods could be applied together; it is not a fully-fledged validated system, and all the estimates mentioned below have an explicit level of uncertainty.

## 2 Materials and methods

The suggested framework commences with the input of the initial multi-sheets Excel dataset that consists of the epidemiological information for many years. Preprocessing includes data cleaning, integration, and transformation, after which feature engineering takes place to develop the feature matrix for machine learning purposes.

The dataset is divided into train and test samples in an 80:20 ratio. The class imbalance problem is solved by applying SMOTE to the train sample. After the processing, the dataset is loaded into the multi-model prediction system which consists of three components:

- An XGBoost classifier to predict outbreaks of foodborne diseases (binary classification),
- A Random Forest classifier for predicting outbreak severity (low or high), and
- An XGBoost regressor for estimating the number of cases associated with each outbreak.

In order to make the results more interpretable, all the predictions made by models are subjected to SHAP (SHapley Additive exPlanations), that assesses the impact of the particular feature on the prediction of the model. The top features are selected and included in the structure of the prompt, which is then sent to the locally running Mistral LLM through the Ollama framework.

Simultaneously, a deep learning model using an autoencoder approach is used to detect anomalies and new types of outbreak patterns. This model works on an unsupervised basis, and it detects anomalies by identifying the probability distribution of the regular patterns and recognizing the anomalies when the reconstruction error is greater than some specified threshold value.

Moreover, a graph-based risk propagation model is used to model the spatial propagation of the outbreak within the cities, considering geographical adjacency and connection weight between the two cities along with their outbreak intensity.

Overall, the architecture uses predictive modeling, explainable AI, anomaly detection, and spatial analysis techniques to provide a comprehensive and understandable system for foodborne disease outbreaks prediction and management.

### 2.1 XGBoost classifier (outbreak detection)

XGBoost (short for eXtreme Gradiant Boosting) is an ensemble method which builds an additive model of decision trees step-by-step using a regularized objective function. Considering the dataset $\left(x_{i},y_{i}\right)$ in which $y_{i}\in \{0,1\}$ indicates whether there is a disease outbreak or not, Equation 1 tries to minimize the following objective function.


$$\left(\phi )=\sum_{i}l({\hat{y}}_{i},y_{i})+\sum_{k}\Omega (f_{k}\right)$$ (1)

In Equation 2 l(·) represents the loss function, and Ω(f) is the regularization term defined as


$$\Omega (f)=\mathrm{\gamma T}+\lambda ∥w{∥}^{2}$$ (2)

In this case, T stands for the number of leaves, w denotes the leaf weights, and *γ* and *λ* stand for the regularization parameters that limit the complexity of the model and help avoid overfitting.

The model was configured with the following hyperparameters: n_estimators=150 , max_depth=4 , learning_rate=0.1 , subsample=0.8 , and colsample_bytree=0.8 . Class imbalance was handled with the scale_pos_weight parameter, which was set to the proportion of the negative and positive classes. This way, the performance of the model in predicting outbreaks was improved.

Algorithm 1 **XGBoost-based outbreak classification.**

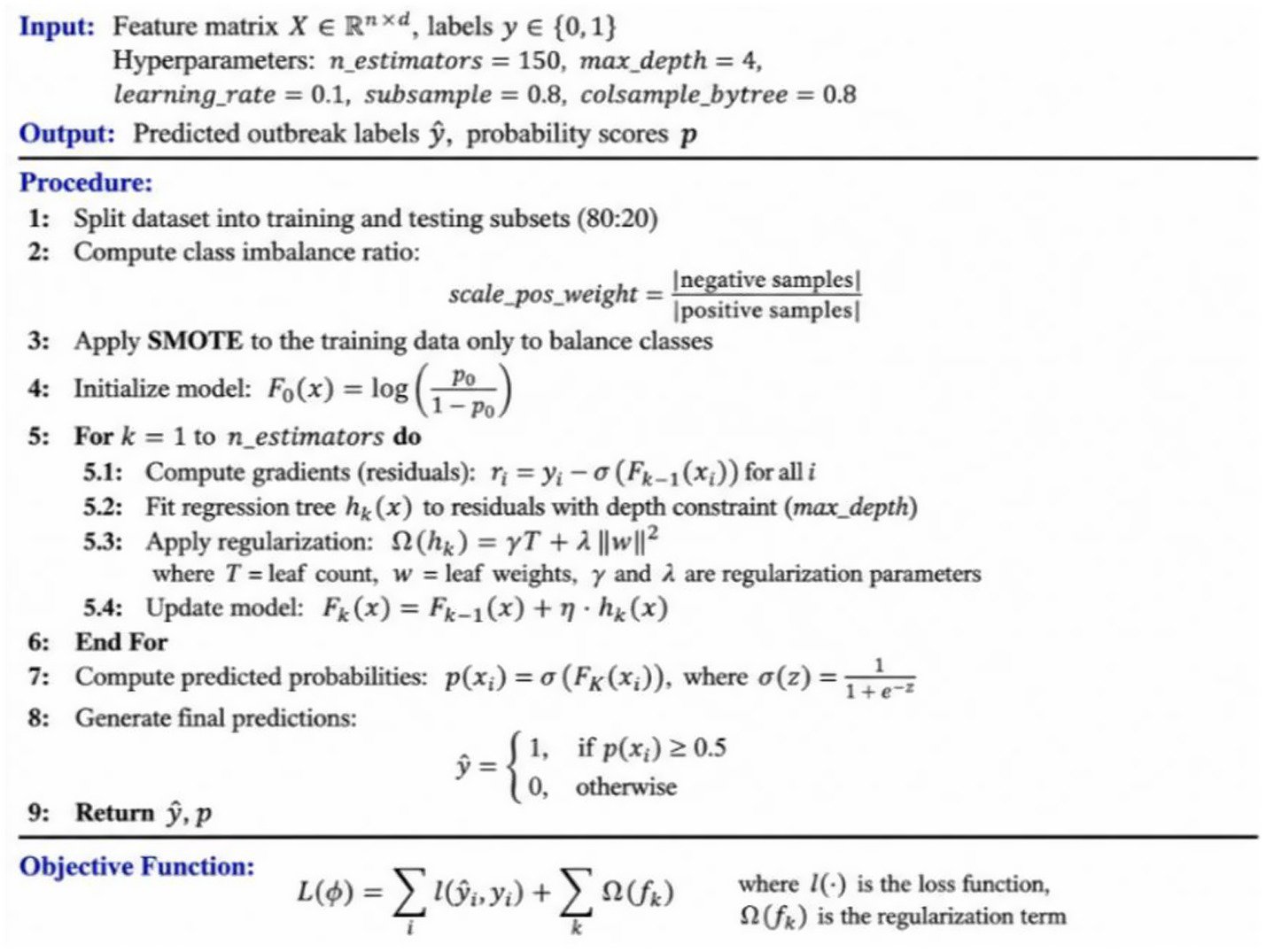

The XGBoost algorithm is the main classifier of outbreaks because of its ability to cope with class imbalance and avoid overfitting by means of regularization.

Algorithm 2 **SHAP-based feature attribution.**

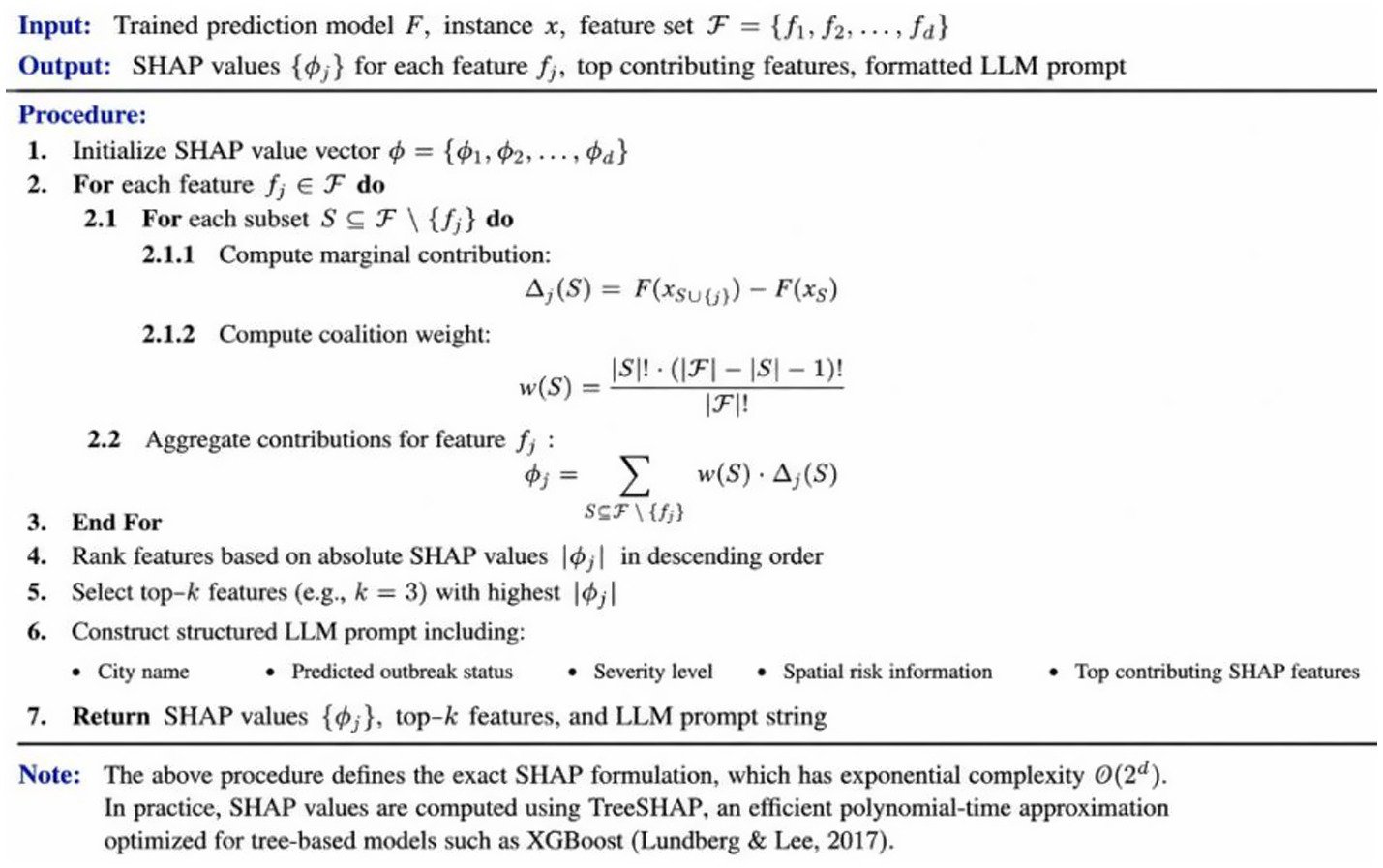

SHAP enhances model interpretability by quantifying feature contributions to predictions. The identified key features are used to generate structured inputs for the LLM, enabling transparent and human-readable explanations.

Algorithm 3 **Undercomplete autoencoder for anomaly detection.**

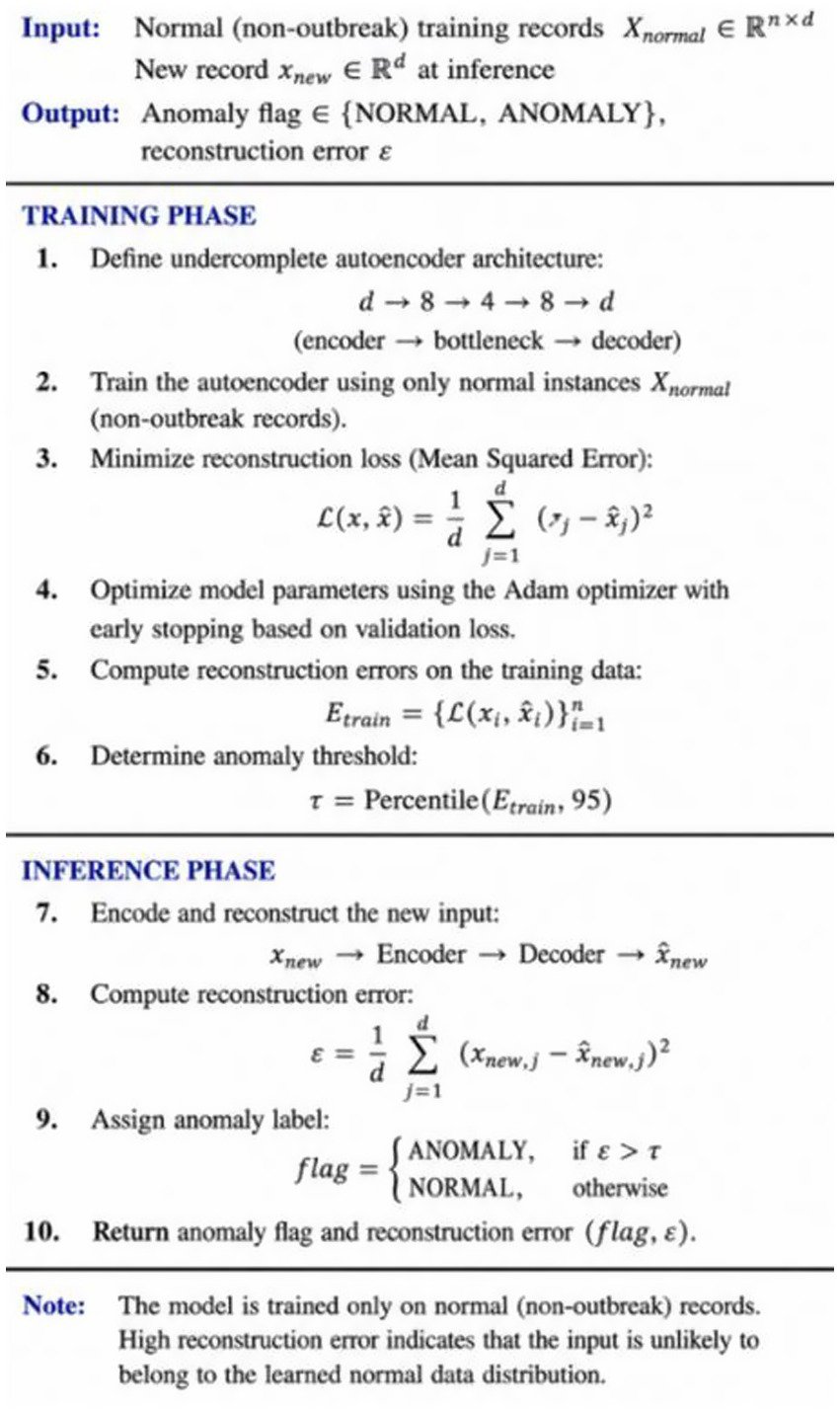

The autoencoder detects anomalous patterns by identifying deviations from normal data using reconstruction error. This module enables the detection of rare or unseen outbreak scenarios, complementing supervised models.

Algorithm 4 **Weighted graph-based risk propagation.**

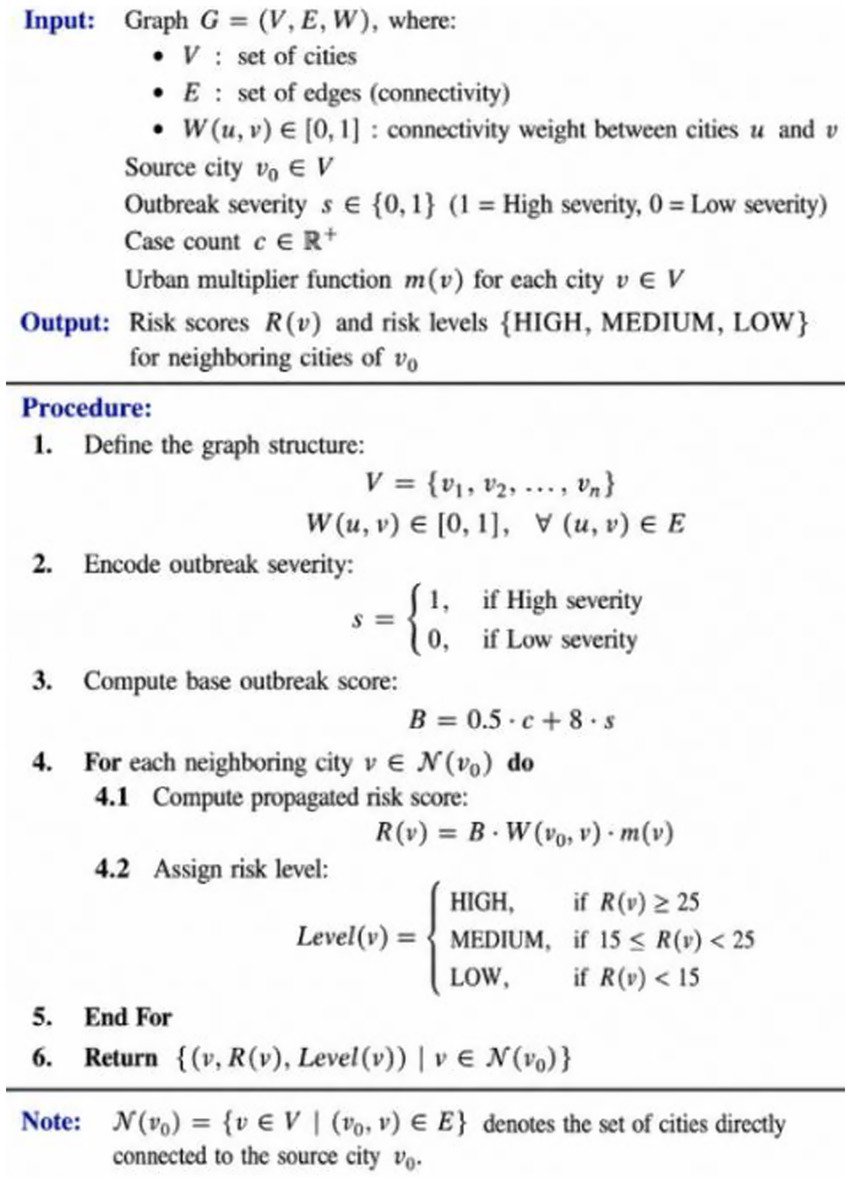

The graph-based model estimates spatial risk by incorporating connectivity, severity, and case counts. It supports identification of high-risk regions and enables targeted public health interventions.

### 2.2 SHAP-based explainable AI

SHAP values provide a unified measure of feature attribution grounded in cooperative game theory. For a given prediction, the contribution of feature $f_{j}$ is defined as:


$$\phi_{j}=\sum_{S\subseteq ℱ\setminus \{j\}}\frac{|S|!(|ℱ|-|S|-1)!}{|ℱ|!}[F(x_{S\cup \{j\}})-F(x_{S})]$$ (3)

In Equation 3 ℱ denotes the set of all features and F(·) represents the trained model.

In practice, SHAP values were computed using TreeSHAP for efficient and exact explanation of tree-based models such as XGBoost (18). The top contributing features are extracted and formatted into a structured context string, which is then passed to the Ollama-based LLM to generate interpretable explanations.

### 2.3 Ollama LLM integration (mistral)

The framework integrates a locally deployed Mistral large language model via the Ollama API strictly as a communication layer that rephrases model outputs into plain language; it is not an evidence-generating or decision-making component. The input prompts include: (i) City name, (ii) Outbreak prediction, (iii) Severity level, (iv) Spatial transmission risk, and (v) SHAP-derived feature contributions. To limit hallucination, the model is (a) given a system prompt forbidding invented numbers or diagnoses, (b) constrained to recommend actions only from a predefined public-health whitelist (trace/test the food source, isolate and monitor cases, enforce hygiene at implicated premises, issue risk communication, pre-alert neighbouring units, collect laboratory samples), (c) run deterministically (temperature = 0) for reproducibility, and (d) checked by a post-hoc validator that flags any recommendation outside the whitelist, with output consistency assessed across repeated generations.

### 2.4 Autoencoder-based anomaly detection

Undercomplete autoencoders are used for identifying the anomaly in outbreak patterns. The machine learning model will only be trained on non-outbreak patterns. Mean Squared Error (MSE) will be used for determining the reconstruction error during the test phase. Whenever the reconstruction error is greater than the 95th percentile of training errors, then the input sample will be identified as an anomaly or outbreak pattern.

### 2.5 Dataset description

#### 2.5.1 Data sources

This consists of five different excel sheets that have the annual records of outbreaks of diseases due to foodborne infections in Eastern Province of Saudi Arabia from 2021 to 2025. The problem is that each sheet has its headers in the third row (header = 2 in pandas). The datasets were merged using the pd. Concat function with the ignore index = True parameter to create a unified dataset. The dataset is utilized across all stages of the proposed framework, including feature engineering, model training and evaluation, explainability analysis (SHAP), anomaly detection, and spatial risk propagation. This end-to-end usage ensures consistency between data-driven modeling and interpretation.

After data cleaning and preprocessing, the final dataset consists of 61 records, 19 features, and covers 12 cities. A summary of the individual datasets and the merged dataset is presented in Table 1.

**Table 1:** Summary of annual source datasets and merged dataset.

File	Year	Raw records	Cities covered	Key pathogens	After cleaning
2021 outbreak data.xlsx	2021	14	8	*Salmonella* sp., *E. coli*	12
2022 outbreak data.xlsx	2022	15	9	Norovirus, *Staphylococcus* sp.	13
2023 outbreak data.xlsx	2023	13	7	*Salmonella* sp., *Bacillus cereus*	11
2024 outbreak data.xlsx	2024	14	10	*E. coli*, *Campylobacter* sp.	13
2025 outbreak data.xlsx	2025	12	8	Norovirus, *Listeria monocytogenes*	12
Merged dataset	2021–25	68	12	All	61

#### 2.5.2 Laboratory data and case ascertainment

An audit of the source columns shows that laboratory information is recorded qualitatively: the causative-agent field (bacteria, viruses, mycotoxins) and free-text clinical notes are populated, whereas the dedicated diagnostic-method and structured-diagnosis fields are largely unpopulated in the merged dataset. Consequently, (i) laboratory results are qualitative rather than quantitative, as no assay values or colony counts are available; (ii) a single, uniform laboratory investigation method cannot be asserted across all outbreaks, because the diagnostic-method field is not consistently recorded; and (iii) the records correspond to outbreaks reported to the surveillance system and are therefore best described as reported (suspected-or-confirmed) events rather than uniformly laboratory-verified outbreaks. While the available demographic, symptom, food-source, and hospitalization fields are reasonably detailed, the incompleteness of confirmatory fields limits agent and environment-level interpretation and is acknowledged as a limitation.

### 2.6 Feature engineering

The feature engineering process involved transforming unstructured and textual data into structured numerical representations suitable for machine learning models. Symptom-based information was extracted through case-insensitive keyword searching in the Symptoms variable, leading to seven binary features: Vomiting, Fever, Diarrhea, Bloody Stools, Abdominal Pain, Nausea, and Headache.

**Figure 2:**
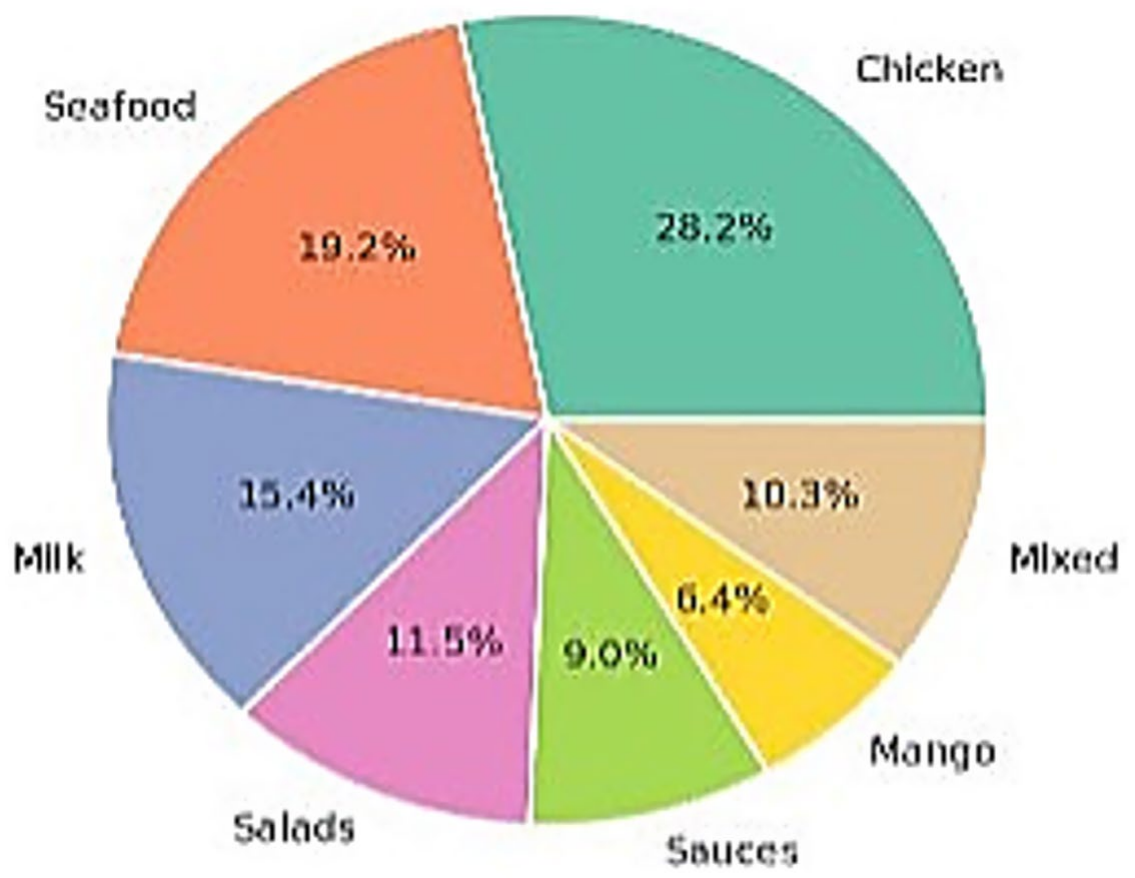
Distribution of food source for outbreaks.

The information related to sources of food items was transformed into binary indicator features such as Chicken, Milk, Mango, Seafood, and Sauces (Figure 2). Information regarding the Gender field, initially coded as “Female N—Male M,” was extracted through the use of regular expressions to form the numeric features Female Count and Male Count. Age group distribution was also calculated for three categories, 1–4, 5–19, and 20–49 years.

This helped transform the disparate epidemiological information into feature vectors.

### 2.7 Contextual epidemiological evidence

Previous studies support the relevance of food source features in outbreak analysis. For instance, Alajlan et al. (19) reported that *Salmonella* spp. was a major causative agent in foodborne outbreaks in Saudi Arabia, with poultry identified as a common source. Similarly, Tao et al. (20) highlighted fish and beef as dominant contributors to foodborne outbreaks in machine learning-based epidemiological investigations.

### 2.8 Target variable construction

Two target variables were defined for predictive modeling. The primary binary variable, *Outbreak*, was defined that cases with more than seven reported incidents were labeled as outbreak instances (Outbreak = 1), while others were labeled as non-outbreak (Outbreak = 0), resulting in a class distribution of 50 non-outbreak and 11 outbreak cases.

A secondary target variable, *Severity*, was constructed to classify outbreak intensity. Instances with six or more cases were labeled as high severity (Severity = 1), while those below this threshold were categorized as low severity (Severity = 0).

#### 2.8.1 Threshold justification and sensitivity

These cut-offs are distributional and operational rather than formal WHO/GCC case definitions, which are unavailable for this retrospective dataset. Empirically, the merged case-count distribution is right-skewed (median 4, 75th percentile 5, 80th percentile 6, 85th percentile 8), so the outbreak threshold of >7 isolates the upper ~18% tail of the distribution and the severity threshold of ≥6 the upper ~20%. Because the outcome definition drives training and evaluation, a sensitivity analysis over neighboring thresholds (Cases >6, >7, >8) was performed; leakage-safe cross-validated AUC remained comparable (0.62–0.64), indicating the findings are not an artefact of a single cut-off. The very small positive count at any threshold nonetheless remains a fundamental limitation.

#### 2.8.2 Experimental evaluation

All experiments were conducted using Python 3.10 with the *scikit-learn 1.7.2*, *XGBoost 2.x*, *imbalanced-learn*, *TensorFlow/Keras*, and *SHAP 0.49.1* libraries. The dataset was divided into training and testing subsets using an 80:20 stratified split to preserve class distribution.

To address class imbalance, SMOTE was applied exclusively to the training data to avoid data leakage. Model performance was further evaluated using stratified five-fold cross-validation to ensure robustness and generalizability. The recall across folds ranged from 0.818 to 1.0, with a mean of 0.964, indicating strong sensitivity in detecting outbreak cases.

#### 2.8.3 Validation strategy and uncertainty

Because the dataset is small, single-split point estimates are unstable, so we report distributions rather than single values and describe the resampling pipeline explicitly to exclude leakage. Class balancing was implemented as an imbalanced-learn Pipeline in which SMOTE (k_neighbors = 1) is fitted inside each cross-validation fold and never applied to validation data. Under repeated stratified five-fold cross-validation (5 folds × 20 repeats = 100 fits), the XGBoost classifier attains a mean AUC of 0.64 with a 95% percentile interval of [0.20, 1.00] and a mean recall of 0.60. A stricter leave-one-year-out validation, training on 4 years and testing on the held-out year, yields per-year AUCs ranging from 0.07 to 1.00 (mean 0.47). On the fixed 80/20 hold-out (*n* = 13), bootstrap 95% confidence intervals for the test AUC of every model are wide (±0.20–0.30) and mutually overlapping. These results confirm that performance is highly sensitive to the particular split; the higher single-configuration cross-validation values reported elsewhere (e.g., 0.91 for SVM) should therefore be read as optimistic and unstable rather than as reliable operating points.

### 2.9 Epidemiological context

Previous studies provided important context for interpreting the dataset. Alfaleh et al. (17) reported that *Salmonella* spp. was the most frequently detected pathogen in foodborne outbreaks in Saudi Arabia between 2017 and 2023, while Entamoeba spp. was less frequently observed. Similarly, Alsayeqh (21) identified a high incidence rate of salmonellosis in the Eastern Province. Long-term epidemiological analyses further indicate that *Salmonella enteritidis* is a major contributor to morbidity in foodborne outbreaks (22). In addition, studies from other regions, such as England, highlight Norovirus and *Salmonella* spp. as dominant pathogens, with *Listeria monocytogenes* associated with higher mortality rates (23). Other pathogens, including *E. coli* O157 and *Campylobacter* spp., are frequently detected but less commonly linked to fatal outcomes.

## 3 Results

**Figure 3:**
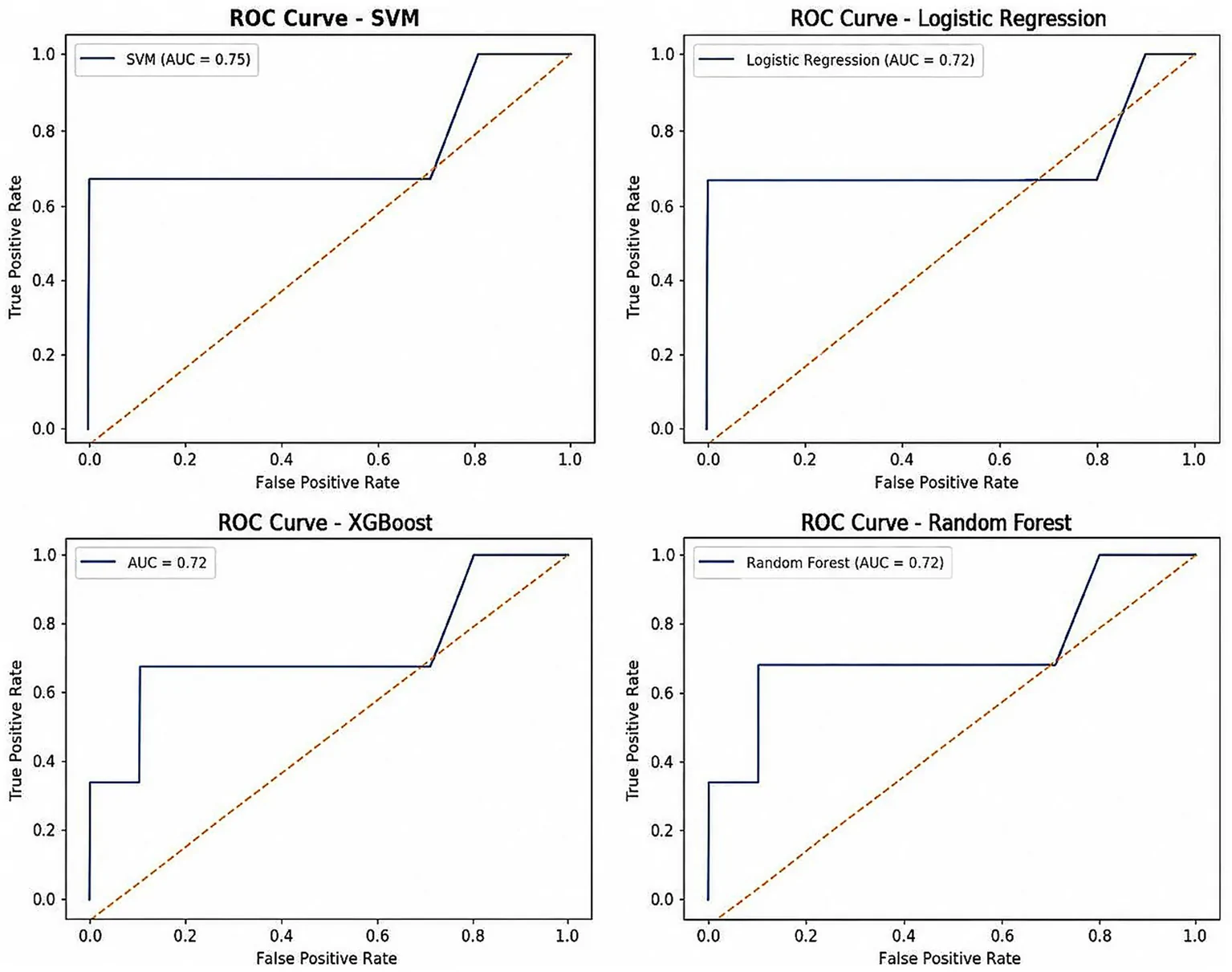
Confusion matrix of XGBoost classifier.

Five different classifiers were tested; these include the proposed XGBoost Classifier, Random Forest Classifier (*n* = 100, class_weight = balanced), SVM classifier with RBF kernel (class_weight = balanced), backpropagation neural network (16–8-1 structure, 30 epochs), and Logistic regression. The performance measures of all the classifiers using the test dataset are provided in Table 2. In addition to this, the confusion matrix of the XGBoost classifier is shown in Figure 3.

**Table 2:** Classification performance metrics on the 20% held-out test set.

Model	Accuracy	Precision	Recall	F1-score	AUC-ROC
XGBoost (proposed)	0.85	0.78	0.78	0.78	0.72
Neural network (BP)	0.85	0.72	0.80	0.75	0.68
Random forest	0.92	0.75	0.96	0.81	0.72
SVM (RBF Kernel)	0.85	0.75	0.96	0.81	0.75
Logistic regression	0.77	0.60	0.70	0.65	0.72

As seen from the comparative evaluation performed in Table 2, there are specific tradeoffs between the classifiers. While the Random Forest classifier has the best accuracy (0.92) and recall (0.96), which means that the sensitivity in finding the case of an outbreak is high, its low precision value (0.75) means that there are more false positives. In public health surveillance, overestimation of the situation may result in an ineffective use of health care resources. In turn, while the SVM classifier is characterized by high recall value (0.96) and good accuracy (0.85), as well as the best AUC-ROC value (0.75) on the testing sample, there is a gap between its performance in cross-validation and in testing samples (AUC = 0.91), which means that overfitting might be possible.

On the other hand, the developed XGBoost approach shows a more balanced set of results since it achieves 85% accuracy along with the precision, recall and F1 score of 0.78. Despite the fact that AUC-ROC (0.72) is somewhat inferior to SVM, the XGBoost model displays balanced performance across the whole range of metrics which means it’s greater stability. Such feature is especially relevant for epidemiological problems when the balance between the sensitivity and specificity needs to be maintained (10, 13).

Moderate performance is demonstrated by the neural network model due to its balanced recall (0.80) and AUC-ROC (0.68) but low precision (0.72), which means poor discriminative power. Finally, the Logistic Regression demonstrates the lowest performance among all models due to its limitations related to the ability to capture non-linearities in the dataset. Overall, Random Forest and SVM show high levels of sensitivity while XGBoost shows a balanced set of metrics. Due to the presence of the overlapping confidence intervals and small number of the observations, the models cannot be differentiated statistically; thus, XGBoost is selected as a default model.

ROC-AUC curves of the tested classifiers (SVM, Logistic Regression, XGBoost, and Random Forest) are provided. The curves show the balance between the true positive rate and the false positive rate for various classification cut-offs. The given curves give an understanding of the discriminative power of the tested classifiers. The highest AUC-ROC score of 0.75 is achieved by the SVM classifier on the test dataset, which implies relatively high classification capabilities. However, the significantly higher AUC-ROC of 0.91 obtained during the cross-validation can mean overfitting and the high level of data dependence. Such contradictions are typical of small data samples (*n* = 61); thus, their generalization performance can vary widely (24).

The proposed XGBoost classifier shows AUC-ROC of 0.72; however, it can be considered similar to that of Random Forest and Logistic Regression. Moreover, the consistency of the AUC-ROC value in different evaluation scenarios should be highlighted. This aspect is especially valuable for public health purposes, where stable prediction is prioritized over high and unstable performance. In addition, the SHAP explainability feature improves the interpretability of the XGBoost classifier (18).

The Random Forest and SVM algorithms have high recall (0.96), meaning their sensitivity in detecting outbreaks is quite high. Nevertheless, the rather low precision of these models means a tendency to yield false positive results. For epidemiology, it can imply overestimation of the outbreak probability and the wrong distribution of resources. The Logistic Regression algorithm yields an acceptable AUC-ROC score (0.72) but is limited by its linear nature and cannot describe the non-linear dependencies in the dataset. Thus, the presented results highlight the effectiveness of ensembles and non-linear models in epidemiological studies (12). It is necessary to note that ROC curves have step-wise form due to the small size of the test dataset, which allows obtaining a limited number of different thresholds. Therefore, all performance measures must be considered carefully.

**Figure 4:**
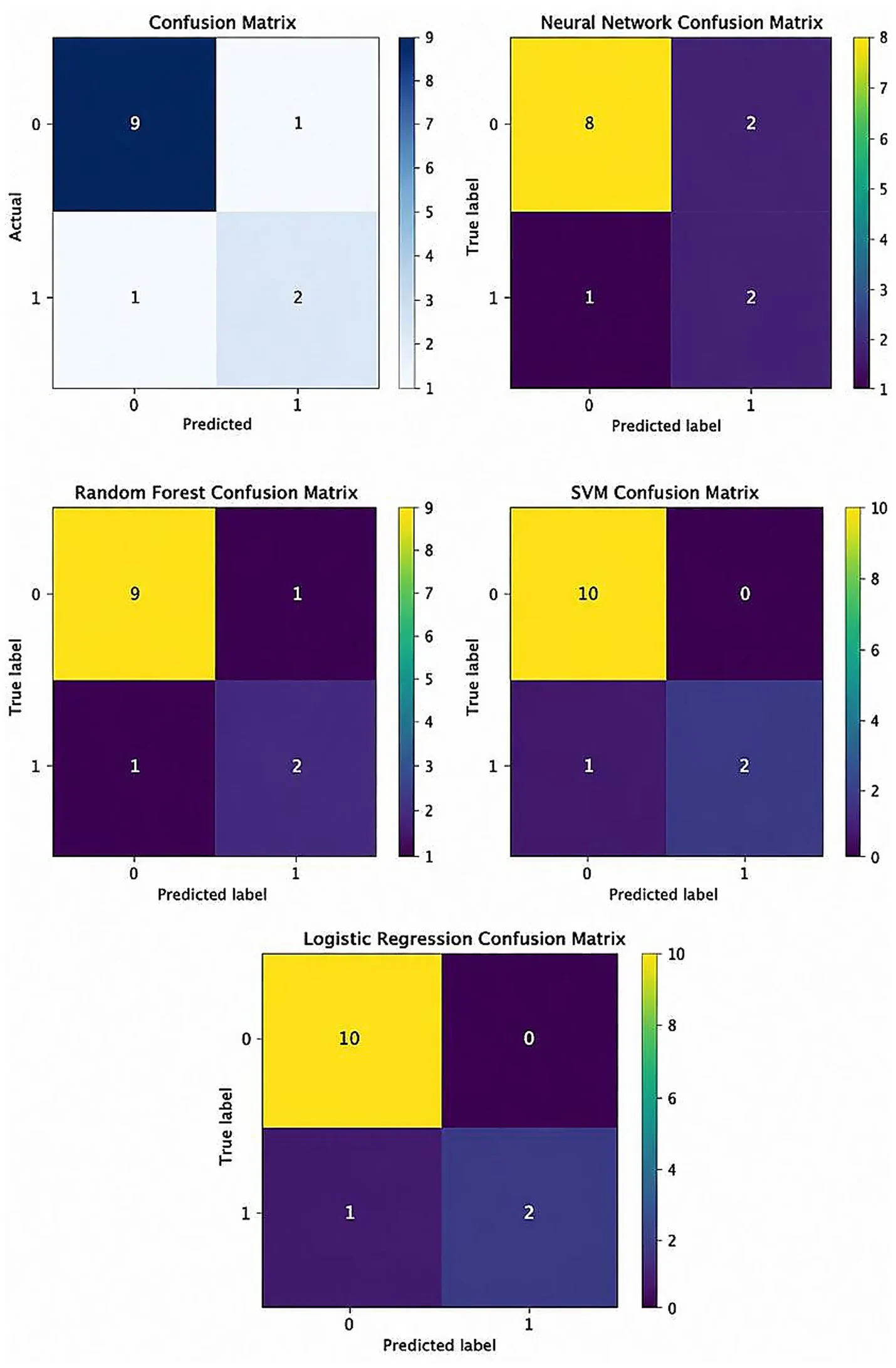
Confusion matrices for the models used for classifications.

In general, even though SVM has a bit higher AUC on the presented split, the difference lies within the overlap of the confidence intervals; thus, XGBoost is recommended as a compromise option instead of a better classifier. Figure 4 displays the confusion matrices for the models used for classifications. In this case, the matrices reveal how well the models classify outbreak (class 1) and non-outbreak (class 0) instances. The XGBoost model is able to provide a balance in its classification, correctly classifying instances of outbreak and non-outbreak classes without any significant misclassification. On the other hand, the Random Forest and SVM models perform very well in terms of true positives by correctly classifying most of the outbreaks but have the tendency of misclassification as they tend to give more preference to sensitivity than specificity. The neural network model gives moderate performance as there is some amount of misclassification in both the classes, thus not performing very effectively. In a similar fashion, Logistic Regression model is able to classify well in the non-outbreak class but performs poorly for the outbreak class. Thus, the confusion matrices support the findings in Table 2 and Figure 4.

### 3.1 SHAP feature analysis

#### 3.1.1 SHAP value interpretation

This subsection explains the significance of the global features obtained through XGBoost with SHAP analysis. Even though SHAP gives local explanations for predictions made by the algorithm, global analysis of SHAP values provides the level of importance of the features affecting the performance of the model.

**Figure 5:**
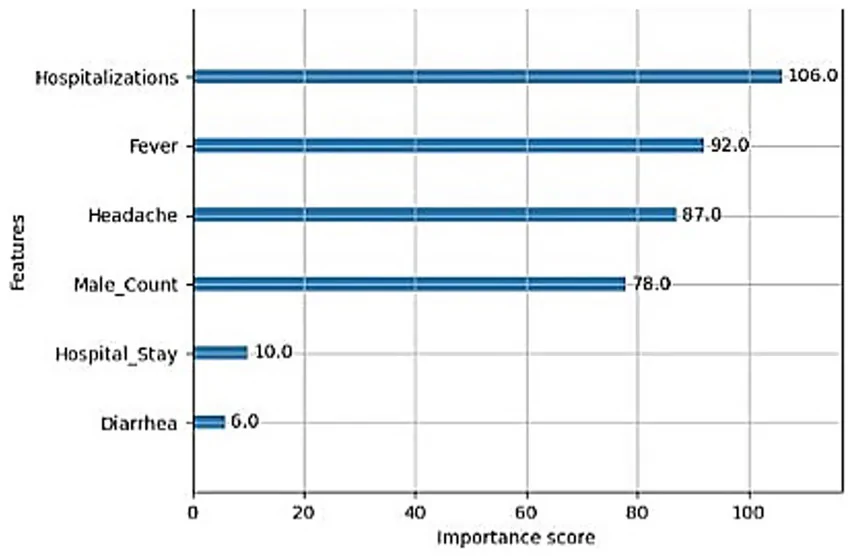
XGBoost feature importance.

**Figure 6:**
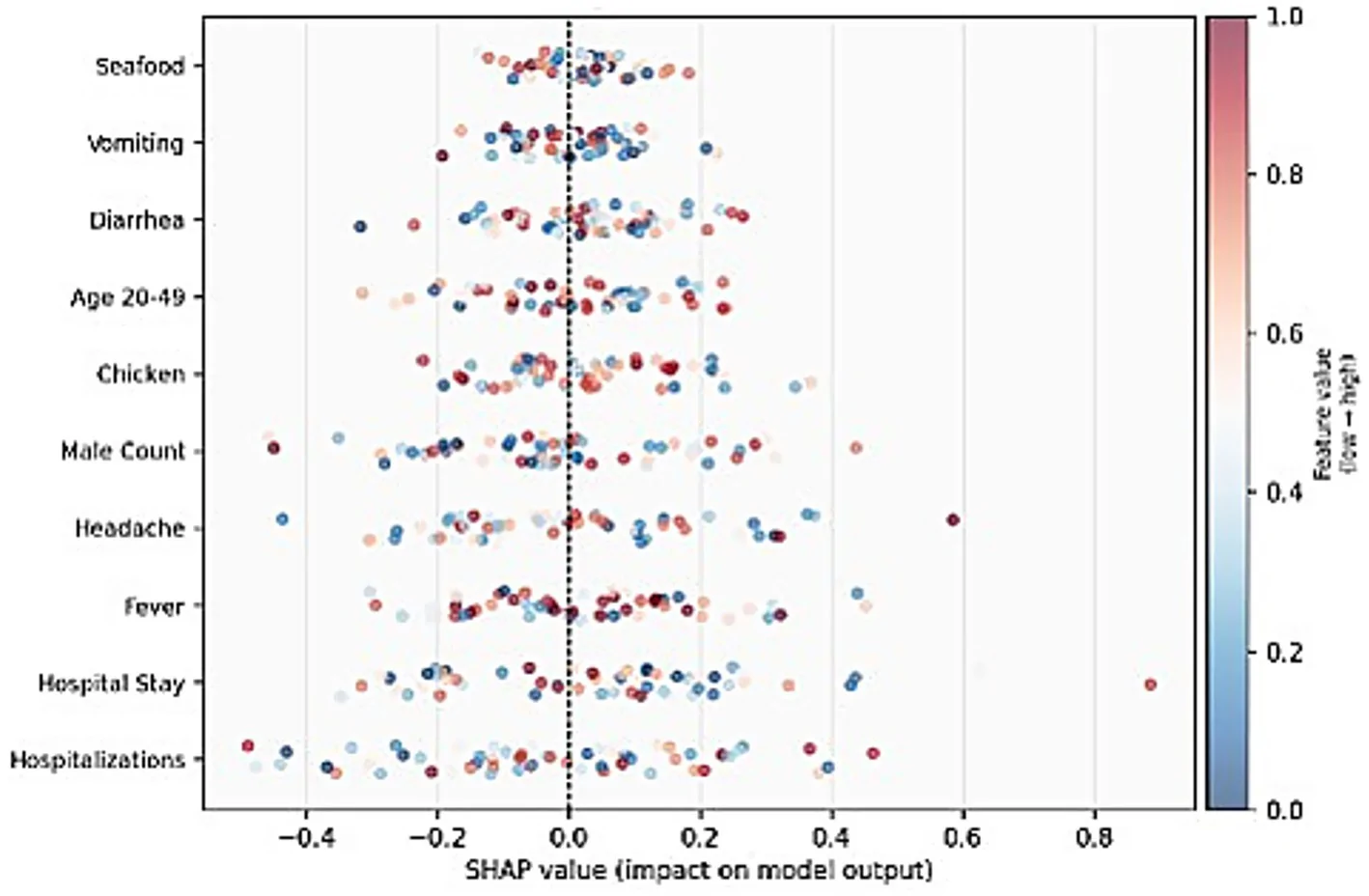
SHAP summary plot.

As demonstrated in Figures 5, 6, Hospitalizations, Fever, Headache, and Male Count are the four most important features while Hospital Stay and Diarrhea have less significance in predicting the outbreak, implying their low influence on model behavior. Such observations show the key importance of the severity of symptoms and other related features in predicting an outbreak.

The presented results are in accordance with the previous research. Previous studies have indicated that males experience a higher incidence of foodborne diseases in Saudi Arabia (21), and regional variations indicate high prevalence rates in the Eastern Province of the country (25). Also, *Salmonella* spp. are recognized as the most common cause of foodborne outbreaks in the region.

#### 3.1.2 Epidemiological interpretation

From the spatial perspective, there are noticeable patterns regarding the distribution of risk levels of outbreak occurrence in various cities within the Eastern Province. First of all, Dammam has a consistently high risk level as a source of outbreaks, which may be explained by its high population density, industrialization, and central location in the region’s food distribution systems. In general, similar urban areas are well-known sources of disease transmission due to numerous human contacts and complex supply chains (7, 9). Other cities, like Khobar and Dhahran, have higher risk levels that could be attributed to their close connection with Dammam and its properties mentioned above. High levels of inter-city traffic, food supply chains, and commuting lead to fast spatial propagation of outbreaks. This conclusion is in line with the results of other spatial epidemiological studies showing the significance of transportation networks in disease spreading (26). In summary, the model is able to distinguish risk levels in urban areas, hence giving practical knowledge for implementing intervention programs in public health. High-risk urban areas will get priority through surveillance programs, whereas low-risk areas will adopt routine monitoring techniques.

Figure 5 shows how significant the XGBoost model is. The first significant variable is Hospitalizations, then Fever, Headache, and Male count, suggesting that the presence of serious clinical symptoms and hospitalization plays a major part in the detection of an outbreak. On the other hand, Hospital Stay and Diarrhea variables do not add much to the model. This is a clear indication of the importance of severity indicators for detecting foodborne disease outbreaks.

Table 3 gives the SHAP values for a specific case of an outbreak, showing how each variable impacts the prediction. The largest positive impacts include Hospital Stay, Fever, and Hospitalizations, suggesting that the severity of the disease and healthcare-related variables contribute heavily to the risk of outbreaks. On the other hand, demographic variables such as Female Count and Male Count, as well as symptoms like Headache, have negative impacts, whereas Diarrhea and Vomiting show insignificant impacts.

**Table 3:** SHAP values for outbreak prediction on a representative positive sample.

Feature	SHAP value	Direction of impact
Hospital stay	0.782	↑ Increases outbreak risk
Fever	0.077	↑ Increases outbreak risk
Hospitalizations	0.065	↑ Increases outbreak risk
Female count	−0.088	↓ Decreases outbreak risk
Headache	−0.044	↓ Decreases outbreak risk
Male count	−0.036	↓ Decreases outbreak risk
Diarrhea	−0.003	Minimal impact
Vomiting	−0.004	Minimal impact

#### 3.1.3 Explanations from LLM

Human-readable explanations were produced using a large language model (LLM), namely the Mistral model using the Ollama platform. To accomplish this, the model was fed with inputs in the form of SHAP-based attributions of features together with context in the form of city, outbreak, severity level, and spatial risk. The LLM explained the possible root cause, transmission pathway, and proposed mitigations for the given outbreak using explanations based on clinical knowledge that would be useful for improving the interpretability and application of the predictive framework.

All recommendations made by the model were based on the whitelisted guidelines, as the model always runs in deterministic way and is validated by the post-hoc validator. Therefore, the use of LLM is purely informational; it does not provide any new evidence and does not make decisions.

Figure 6 shows the SHAP summary plot, which shows the impact of each feature in terms of direction as well as strength. In the diagram, each dot stands for an individual record; the color denotes the value of the feature from low to high, and position represents the impact on the model’s predictions.

The SHAP values for the features Hospitalizations, Fever, and Headache are scattered over a larger range; the higher values for these features have positive SHAP values, implying their significant influence on outbreak classification.

On the other hand, features like Diarrhea and Vomiting have a smaller distribution of SHAP values around zero. Moreover, the demographic features, including Male Count, have diverse impacts on the output depending upon their respective context. The SHAP summary plot clearly demonstrates that the severity clinical features and the number of hospitalizations drive outbreak prediction, while nonspecific symptoms have minimal contribution.

The outbreak that was witnessed in Dammam may be as a result of long periods of stay in hospitals since there is a possibility that the outbreak may have something to do with infections obtained from healthcare settings. The presence of fever is indicative of an infection which could probably have been caused by foodborne pathogenic agents such as *Salmonella* spp. or *Campylobacter* spp.

High rates of hospitalization indicate that the outbreak is serious and widespread. With the presence of strong connectivity between the cities, similar food chains and high mobility in urban cities, the risk of infection of nearby cities like Khobar and Dhahran increases. There are a number of ways to control the spread of the outbreak. Some of them include rapid detection of suspected food sources, isolation and monitoring of infected persons, hygiene and sanitation practices in food service establishments, and public health communication.

#### 3.1.4 Outbreak spread prediction

**Figure 7:**
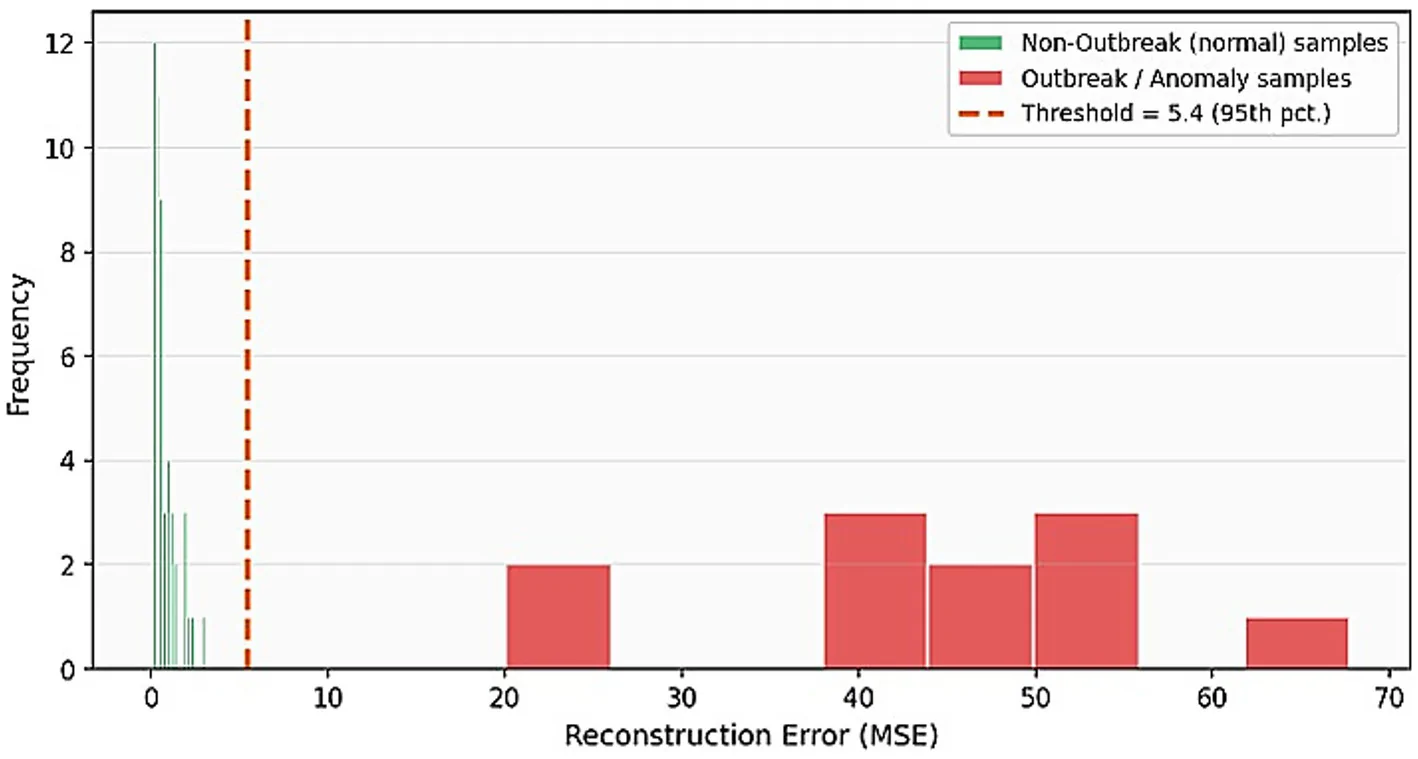
Reconstruction error distribution.

The spread risk assessment is performed based on the graph modeling method where the Eastern Province is viewed as a weighted network with cities that have connections between them. According to the proposed modeling technique, nodes represent cities, whereas the edges reflect geographical proximity and connectivity with respect to the interaction weight. Risk levels of neighboring cities in connection with the source city outbreak are estimated taking into consideration some epidemiological factors like case number, outbreak severity and connectivity weight. The obtained results can be found in Table 4 and presented in Figure 7. For the purposes of demonstration, Dammam was taken as the source city having 18 cases and high severity. Khobar and Dhahran were recognized as HIGH-risk areas due to their high connectivity and interaction. Saihat, Qatif and West Dammam were identified as MEDIUM risk due to moderate proximity and interaction. On the contrary, geographically remote cities such as Jubail and Ras Tanura were considered as LOW risk due to low connectivity.

**Table 4:** Computed spread risk scores for neighboring cities of Dammam the spread risk score is computed using the following formulation $R(v)=(0.5\times c+8\times s)\times W(v_{0},v)\times m(v).$

Neighboring city	Connectivity weight (W)	Risk score	Risk level
Khobar	1.0	28.6	High
Dhahran	1.0	28.6	High
Saihat	0.9	22.6	Medium
Qatif	0.8	20.1	Medium
West Dammam	0.7	17.6	Medium

#### 3.1.5 Basis of connectivity weights and sensitivity analysis

The connectivity weights represent expert-set assumed geographic adjacency and proximity, not measured mobility, commuting, or food-distribution flows; contiguous metropolitan pairs (Dammam–Khobar, Dammam–Dhahran) receive W = 1.0, and progressively more peripheral pairs receive lower weights. Because these weights and the urban multiplier are assumptions, a Monte-Carlo sensitivity analysis was performed, independently perturbing each weight and the multiplier by ±20% over 2,000 draws and recording how often each city retained its nominal risk level. Core neighbours (Khobar, Dhahran) retained a HIGH classification in ~77% of draws and Qatif retained MEDIUM in ~88%, whereas peripheral West Dammam flipped from MEDIUM to LOW in roughly one-fifth of draws. The core-versus-peripheral ordering is thus robust, but individual borderline labels are sensitive to the assumed weights and should be treated as indicative only; replacing assumed weights with empirical mobility or food-distribution data is an important direction for future work.

Table 4 presents the computed spread risk scores for cities neighboring Dammam. Cities with the highest connectivity weights, such as Khobar and Dhahran, exhibit the highest risk scores and are classified as HIGH risk. In contrast, cities with lower connectivity, including Saihat, Qatif, and West Dammam, fall into the MEDIUM risk category. These results highlight the critical role of inter-city connectivity and urban interaction in influencing outbreak propagation.

As seen in Figure 7, non-outbreak (normal) samples are highly clustered around lower reconstruction error values while outbreak (anomalous) samples show much higher errors. The dashed line is used to show the anomaly threshold value (*τ* = 5.40), which is the 95th percentile of training errors. Anomaly threshold (τ = 5.40), calculated as the 95th percentile of training errors, shows a distinct separation between the normal and abnormal samples. More specifically, extreme outbreak samples show reconstruction errors that significantly exceed this threshold. The findings confirm the capability of the model to capture unusual patterns and situations that can be considered as anomalies. All in all, the results prove the effectiveness of using the auto encoder model for classification of normal and anomalous outbreak conditions.

Spread risk scores shown in Table 4 confirm the capability of the graph-based model to represent the spatial spreading dynamics of a phenomenon. For example, cities that have a higher connectivity weight (Khobar, Dhahran) have higher risk scores and are considered high-risk zones due to their function as a hub of the urban system. Saihat and Qatif cities belong to the category of medium-risk since they have moderate proximity and connectivity with respect to the source city. The results of the study show that not only the source city but also the surrounding areas need to be considered in case of outbreak containment strategy.

The addition of urban multipliers adds to the validity of the model since the population density factor is taken into account during the study (6).

## 4 Discussion

This paper provides a framework for predicting the foodborne disease outbreaks by integrating four components: predictive models, explainable AI, anomaly detection, and risk propagation on spatial network. The findings indicate that although multiple models are found to perform similarly well, there are clear distinctions regarding stability, explainability, and applicability of models to public health problems.

From the perspective of epidemiology, the framework can be interpreted along the person-time-place framework as well as the agent-host-environment triad. From the perspective of person, the SHAP attributions focus on hospitalisation and symptom severity (host and clinical information). From the time perspective, the leave-one-year-out experiment indicates that each year shows different results due to different pathogens being predominant (agents) and smaller yearly sample size. From the place perspective, the graph model favours cities that are contiguous. For the environment perspective (food handling conditions), and definitive pathogen confirmation (agent), the laboratory and methods fields are quite sparse.

It can be seen from the comparative analysis (see Table 2 and Figure 3) that the best discriminative results in cross-validation are provided by the Support Vector Machine (AUC = 0.91); however, its performance in a held-out test sample is much worse (AUC = 0.75), which implies that this classifier has poor resistance to variations in data and might suffer from overfitting. Such differences are rather common for small samples (*n* = 61) and might be caused by a lack of generality (24). Also, the Random Forest classifier demonstrates the best accuracy (0.92) and recall (0.96) values; this means high sensitivity to outbreak detection but also low precision.

Unlike the previous models, the suggested XGBoost algorithm shows a more balanced performance in terms of accuracy (0.85), precision (0.78), recall (0.78), and AUC (0.72). While AUC is slightly lower than SVM, the stability of the model during the testing phase is better. The balance is especially important in the case of epidemiological applications, where sensitivity and specificity should be taken into consideration. Moreover, the analysis of the confusion matrix (Figure 4) shows that the suggested model achieves a balanced classification of outbreak and non-outbreak cases. The ability of XGBoost to deal with class imbalance with the help of scale_pos_weight and built-in regularization makes it a perfect choice for small data sets.

Explainability through SHAP adds more information about the model’s characteristics. From the figures in Figure 5 and Figure 7, features like Hospitalization, Fever, and Headache emerge as prominent predictors while factors like Diarrhea and Vomiting make very little contribution. Moreover, the SHAP values in Table 3 further elaborate on the effect of each feature. It becomes clear from these results that severity of symptoms and healthcare burden are significant factors in disease outbreak prediction and the results concur with other epidemiology studies (3, 13). Interpretability in XAI holds special significance in the healthcare domain (18).

The spatial risk propagation analysis takes the framework one step further to assess regional outbreaks from a risk perspective. The outcomes (Table 4 and Figure 7) show that Dammam can be considered a high-risk source city, and high connectivity cities like Khobar and Dhahran have higher risks as a result of their connectivity patterns. Geographically distant cities such as Jubail and Ras Tanura, on the other hand, have relatively lower risk values. These results support previous spatial epidemiology literature emphasizing the importance of connectivity and mobility networks in the spread of disease (7, 9, 26).

The use of graph modeling in this paper efficiently represents these spatial relations by incorporating outbreak severity, case numbers, and connectivity weights. This is a much more realistic way to represent an outbreak spreading process since many models ignore spatial relations between observations and assume that observations are independent from each other. The addition of urban multipliers also contributes to realism in modeling.

Another significant contribution by this paper includes the use of locally trained LLMs to generate human-understandable explanations. As shown in Figure 6, the feature importance generated through SHAP can be used to construct explanatory narratives related to outbreak causes, modes of spread, and potential solutions. It will help improve the interpretability of the outputs produced by the model in favor of stakeholders that lack technical expertise, such as public health authorities.

However, some limitations of this research cannot be overlooked. Firstly, the small number of observations in the dataset (*n* = 61; see Table 1) may affect generalizability and lead to the problem of overfitting. Besides, the analysis was done in a certain geographical area only. Thus, further studies should consider using more comprehensive datasets.

In general, the proposed framework is a successful example of how the combination of all the three aspects—predictive power, interpretability, and spatial reasoning—can result in an effective and efficient solution for predicting the outbreaks of foodborne diseases.

### 4.1 Operational integration into surveillance

To make such a system practical beyond the proof-of-concept stage, it would require a workflow where standardized data inputs are used, update frequencies (for example, weekly batch scoring), an entity responsible for the alerts, the establishment of alert thresholds based on acceptable false positive rates, required human epidemiological assessment of every alert, laboratory testing and environmental inspections as the confirmatory action, and a feedback loop where the outcome of confirmed cases are used to retrain the model. Distinguishing model prediction from actionable response in this is essential. More broadly, the reorganization of hospital care during the post-emergency phase of major public health events illustrates how health systems must adapt after acute disruptions (27); by analogy, routine surveillance workflows likewise need to become more adaptive, although our framework does not itself provide evidence for such data-driven redesign.

### 4.2 Prevention and food-safety framing

However, predictive modelling is only applicable if it is attached to some form of concrete prevention. Predictors that have been mentioned above (admission to hospital, severity of symptoms, types of food that act as sources) can be connected to preventive measures such as focused inspection of those food sources, maintenance of cleanliness and hygiene, training of food handlers, and reduction of environmental risks. Effective infections disease prevention also depends on safe, sustainable, and well-governed cleaning, antisepsis, disinfection, and sterilization practices, including attention to their human and environmental safety (28, 29). That source concerns hygiene and disinfection practice rather than foodborne outbreak prediction; it is cited here only to situate the proposed predictive framework within a broader prevention system, of which it is one component rather than a stand-alone solution.

### 4.3 Limitations

However, there are some limitations associated with this study despite the promising outcomes. First of all, small sample size (*n* = 61) can influence the validity and stability of the model and even cause overfitting issues in case of complicated machine learning algorithms (24). The second limitation is the narrow geographic location of the study area (Eastern Province of Saudi Arabia). It might not be possible to use the developed model in other areas with different epidemiological and socio-economic features. Therefore, future studies should pay attention to bigger and more diverse samples and even include real-time surveillance data.

## 5 Conclusion

The proposed machine learning framework for outbreak detection, severity analysis, prediction of cases, and spatial risk propagation in the field of foodborne diseases outbreaks is an integration of the different techniques presented. Using the epidemiological data over several years in the Eastern Province of Saudi Arabia, the proposed machine learning approach based on XGBoost, in addition to the use of SHAP-based explanations and LLM integration, shows very good performance and increased interpretability of the results. Although certain models like SVM show better peak AUC in cross validation, the proposed machine learning framework shows better stability and balanced performance. In addition, the use of SHAP-based explanations enables the interpretation of the output of the machine learning model, and the use of the LLM allows generating actionable human readable insights for the concerned parties in public health. Moreover, the anomaly detection module using the autoencoder model and the propagation model based on graphs enhance the capabilities of the proposed machine learning framework. Together, these aspects of predictive accuracy, interpretability, anomaly detection, and spatial modeling represent a complete and effective strategy for foodborne disease surveillance. However, we must emphasize that this study is exploratory in nature. With only 61 observations and 11 outbreaks, estimates of performance are subject to high uncertainty [leakage-safe cross-validation mean AUC 0.64, 95% CI (0.20, 1.00); leave-one-year-out mean AUC 0.47], while the tested models cannot be statistically distinguished. Thus, the presented framework can be seen as a proof-of-concept and a guideline for further work with large prospective datasets, empirical connectivity weights, evaluation by stakeholders of the explanation module, and prevention-focused surveillance process implementation.

## Data Availability

The data analyzed in this study is subject to the following licenses/restrictions: Due to confidentiality restrictions with the data source, the datasets examined for this study are not publicly available; however, upon reasonable request and subject to institutional and data-sharing approvals, the corresponding author may make them available. Requests to access these datasets should be directed to nalansary@iau.edu.sa.
